# Accumulation and Biotransformation of *Dinophysis* Toxins by the Surf Clam *Mesodesma donacium*

**DOI:** 10.3390/toxins10080314

**Published:** 2018-08-04

**Authors:** Juan Blanco, Gonzalo Álvarez, José Rengel, Rosario Díaz, Carmen Mariño, Helena Martín, Eduardo Uribe

**Affiliations:** 1 Centro de Investigacións Mariñas, Xunta de Galicia, Pedras de Corón S/N, 36620 Vilanova de Arousa, Spain; maria.carmen.marino.cadarso@xunta.gal (C.M.); helena.martin.sanchez@xunta.gal (H.M.); 2 Departamento de Acuicultura, Universidad Católica del Norte, Larrondo 1281, Coquimbo, Chile; jrengel@unc.cl (J.R.); rdiaz@ucn.cl (R.D.); euribe@ucn.cl (E.U.); 3 Centro de Investigación y Desarrollo Tecnológico en Algas (CIDTA), Facultad de Ciencias del Mar, Larrondo 1281, Universidad Católica del Norte, Coquimbo, Chile

**Keywords:** pectenotoxins, surf clam, accumulation, biotransformation, depuration

## Abstract

Surf clams, *Mesodesma donacium*, were shown to accumulate toxins from *Dinophysis acuminata* blooms. Only pectenotoxin 2 (PTX2) and some of its derivatives were found, and no toxins from the okadaic acid group were detected. PTX2 seems to be transformed to PTX2 seco-acid (PTX2sa), which was found in concentrations more than ten-fold those of PTX2. The seco-acid was transformed to acyl-derivatives by esterification with different fatty acids. The estimated amount of these derivatives in the mollusks was much higher than that of PTX2. Most esters were originated by even carbon chain fatty acids, but some originated by odd carbon number were also found in noticeable concentrations. Some peaks of toxin in the bivalves did not coincide with those of *Dinophysis* abundance, suggesting that there were large differences in toxin content per cell among the populations that developed throughout the year. The observed depuration (from the digestive gland) was fast (more than 0.2 day^−1^), and was faster for PTX2 than for PTX2sa, which in turn was faster than that of esters of PTX2sa. PTX2 and PTX2sa were distributed nearly equally between the digestive gland and the remaining tissues, but less than 5% of the palmytoyl-esters were found outside the digestive gland.

## 1. Introduction

Toxins produced by the dinoflagellate genus *Dinophysis* frequently accumulate in bivalves making them unsafe for human consumption and leading to closures of fisheries or marketing of aquaculture products. The impacts of these toxins are widely distributed across the oceans, but some areas are particularly affected, as is the case in Southern Chile and North-Western Spain [1,2,3,4,5,6,7,8].

**Figure 1:**
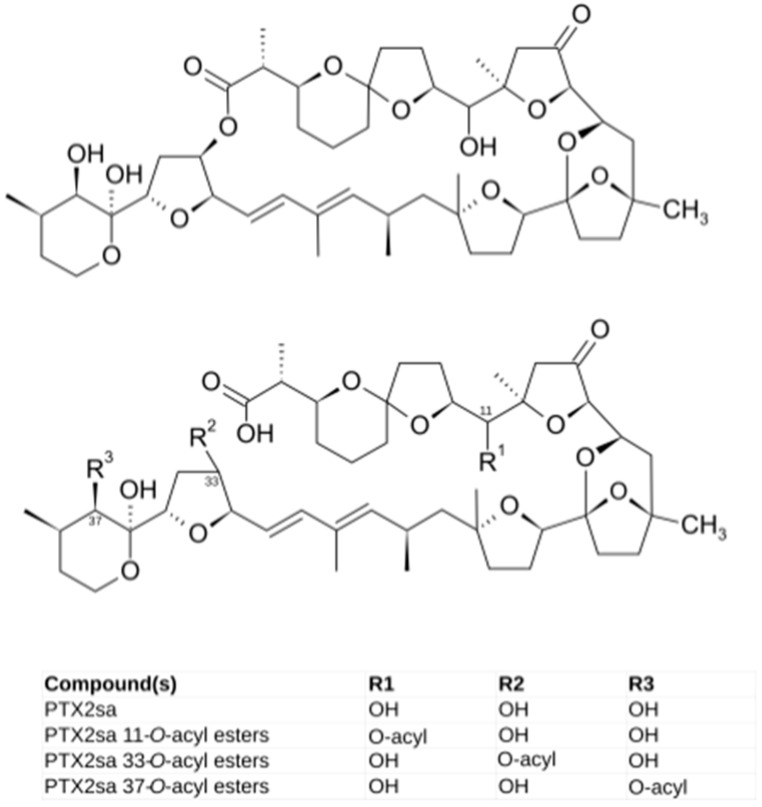
PTX2 (upper structure) and PTX2 seco-acid (PTX2sa) and its acyl esters (lower structure).

Species of the genus *Dinophysis* are known to produce two different groups of toxic compounds: toxins of the okadaic acid (OA) group and pectenotoxins (PTX) [7]. The production of one or both types of toxins is known to be species-specific, but important strain variation exists. Some species produce only pectenotoxins (Figure 1) while others usually produce toxins of both groups, although in some cases, with a low relative proportion of pectenotoxins [7]. While the toxins of the OA group have caused numerous intoxications [9], there is no evidence that PTXs are toxic for humans by oral exposure [10]. However, due to their toxicity by intraperitoneal injection (and some contradictory results about the effects of oral administration) in mice and rats, some regulatory systems, such as the European one, still maintain quarantine levels for these compounds [11,12], with a noticeable incidence for products that target these markets.

In many bivalves, the accumulated toxins of the okadaic acid (OA) group are transformed to 7-*O*-acyl derivatives (generically known as DTX3) by esterification with fatty acids of different carbon chain length [13,14,15]. Very likely this is the main route for the elimination of those compounds from the bivalves. Less information exists for pectenotoxins, but it is known that they can be enzymatically transformed to their corresponding seco-acid (by opening the macro-ring of the molecule) in the digestive system of some mollusks [16]. These seco-acids can be esterified by fatty acids (as in the case of the toxins of the OA group) at least in the mussel *Mytilus edulis* [17] and in an Australian clam (probably *Plebidonax deltoids*) [14], suggesting that this can also be a depuration route.

In the northern region of Chile, the impact of the toxins produced by *Dinophysis* is less than in the south, but some closures, mostly of the economically important aquaculture of the pectinid *Argopecten purpuratus*, have taken place, as happened in 2005 due to a bloom of *Dinophysis acuminata* [18]. In that case *D. acuminata* was shown to have an atypical toxin profile, producing only pectenotoxins, without traces of toxins of the okadaic acid group. *D. acuminata* had been shown to be present in the north of Chile many years earlier [19,20,21], and could be assumed to be persistent in the area. DSP harvesting closures in the area, notwithstanding, were not needed until October 2005 [18], suggesting that toxin production was low, or that the toxins produced were quickly degraded or depurated from the bivalves in the area.

In this work, we studied *D. acuminata* populations, and the accumulation in the surf clam *Mesodesma donacium* of the toxins produced by this species in Coquimbo Bay, a significant fishing area for this economically important species. The objectives of the study were: (a) to obtain the profile of accumulated toxins; (b) to check if the accumulated toxin follows the *D. acuminata* cell abundance; (c) to obtain an estimate of the depuration rate of the toxins involved; and d), to gather knowledge about the possible transformations that take place in the bivalve.

## 2. Results

### 2.1. Abundance and Composition of Dinophysis Populations

**Figure 2:**
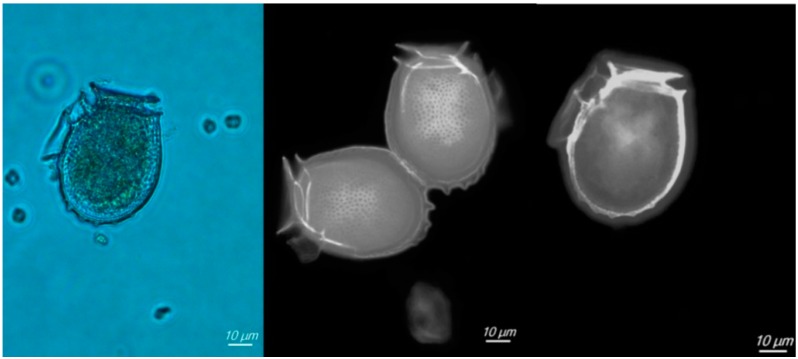
Phase contrast (**left**) and fluorescence photomicrographs of Calcofluor stained (**right**) *Dinophysis acuminata* cells from samples of the study.

*Dinophysis* populations were always present in the area and were dominated by *Dinophysis acuminata*. Its abundance was generally low, with cell concentrations below 300 cells L^−1^ in 75% of the sampled weeks. However, some blooms, with abundances over 900 cells L^−1^ were recorded in April 2009, and in January and February 2010, reaching a maximum of 2100 cells L^−1^. On some occasions, *Dinophysis caudata* and *D. tripos* were detected but only in net samples (with very low concentrations) and their populations could not be quantified. The cells of *Dinophysis acuminata* were almost oval in shape with the left sulcal list well developed and extending about one-half to two-thirds of the cell length (Figure 2). The thecal plates that constitute the hypotheca were covered with circular areolae. The antapex of the cells was rounded, and in some cells two to four small knob-shaped posterior protrusions were found. The length (L) of the cell was 47.61 ± 3.87 μm and the dorso-ventral width (W) was 34.69 ± 3.47 μm, while the L/W ratio was 1.38.

### 2.2. Toxin Profiles

**Figure 3:**
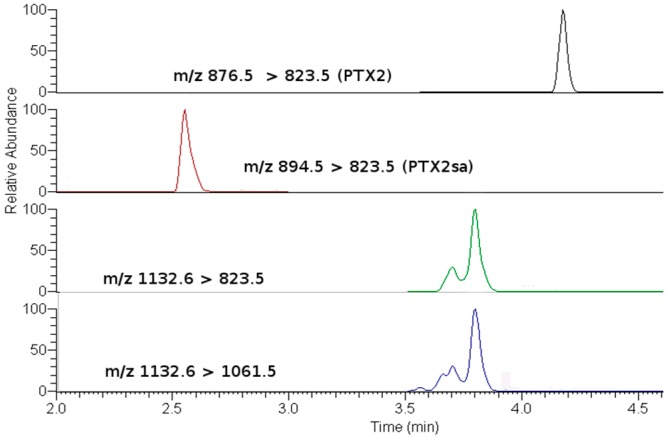
Chromatograms of the main pectenotoxin (PTX) analogs detected (sample of the digestive gland on 12 August 2009). The two lower chromatograms correspond to transitions of palmytoyl-esters of PTX2sa. The upper one of these is more affected by C33 and C37 esters and the lower one of these is also affected by C11 esters.

**Figure 4:**
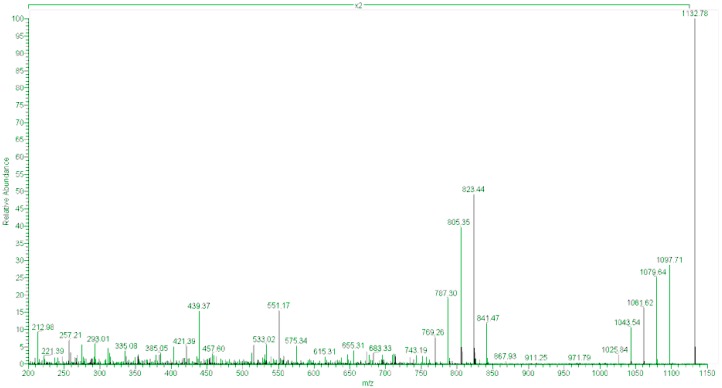
Fragmentation spectrum of palmitoyl-PTX2sa (main peaks).

**Figure 5:**
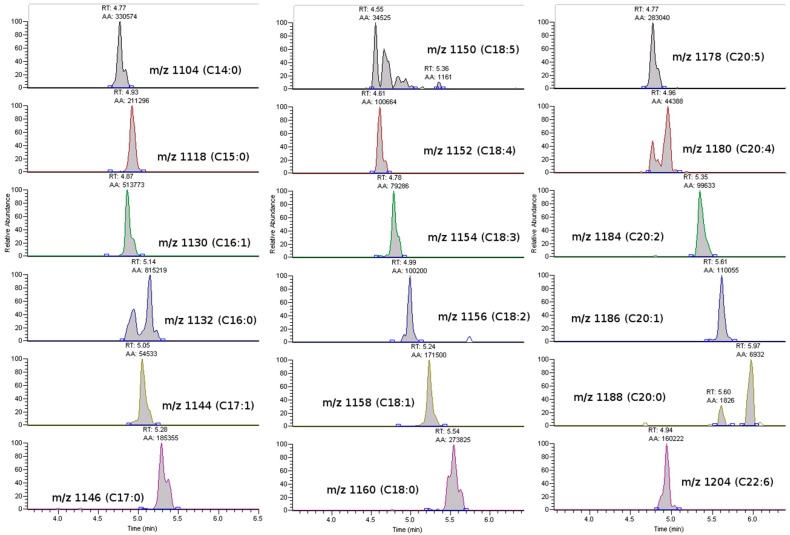
Chromatograms of the main acyl-derivatives of PTX2sa in the sample corresponding to the digestive gland of the *Mesodesma donacium* taken on 12 August 2009. *m*/*z* numbers are the parent masses (product *m*/*z* = 823) corresponding to esters of PTX2sa with fatty acids of the indicated chain.

OA, DTX1 or DTX2 were not detected in either the raw or the hydrolyzed samples, in this study. The only PTX found was PTX2, which was accompanied by its seco-acid and by acyl-esters of its seco-acid (Figure 3 and Figure 4). None of the other monitored PTX compounds (Table 1) were found. The main esters of PTX2-sa found were produced by esterification with palmitic acid (C16), but other esters—from fatty acids with even carbon numbers (mainly C16:1, C14:0, C18:0, C18:1, C10:5) and with odd carbon numbers (mainly, C15:0, C17:0 and C17:1)—were also found (Figure 4). The detected acyl-esters seem to be mostly products of the esterification of the hydroxyl groups at C33 and/or C37, as their fragmentation pattern presented relevant peaks at *m*/*z* 823, which is typical of these types of esters (Figure 5). Additional small peaks also appeared when the product *m*/*z* 1061.5 was monitored, probably due to the presence of C11 esters. A regression of the signals of *m*/*z* 823 and 1061 in all measured samples gave an R^2^ of 0.999, indicating that the contribution of the C11 esters (associated with *m*/*z* 1061 but not with *m*/*z* 823) was very small.

Several esters, and perhaps several conformational isomers of them, for each fatty acid may be involved as they were not resolved as a unique chromatographic peak, as shown for the esters with palmitic acid (Figure 3). 

PTX2 seco-acid (PTX2sa) concentrations were much higher than those of PTX2. Even though the precise contribution of PTX2sa could not be determined because of the lack of reference solutions, the response in our method (estimated by means of a biotransformation experiment not reported here) was approximately one-third that of PTX2. Taking this into account, the PTX2sa concentrations found were on average nearly 20-fold and 10-fold those of PTX2, in the digestive gland and in the remaining tissues, respectively.

**Figure 6:**
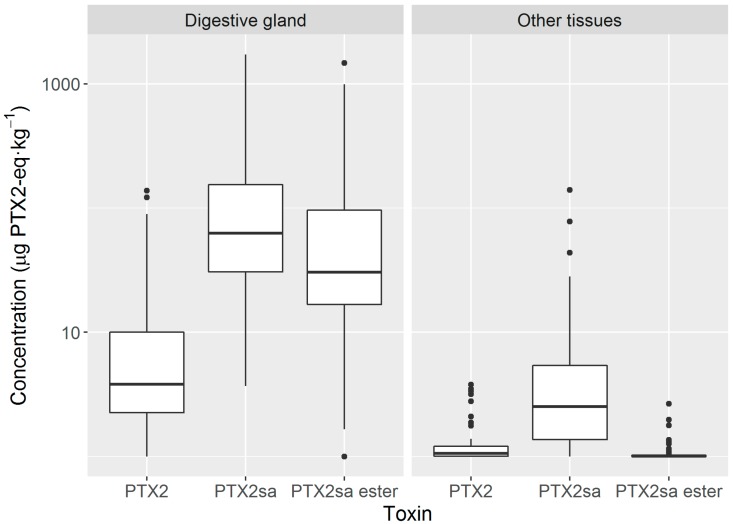
Concentration of the studied toxins in digestive gland and remaining tissues of *Mesodesma donacium* (PTX2sa ester = palmytoyl-PTX2sa). The limits of each box correspond to the 75% and 25% quartiles. The central horizontal line inside the box is the median. The extremes of the vertical lines are the extreme observations excluding the outliers and the isolated dots are outliers.

Assuming that the response of the palmitoyl-esters of PTX2sa detected in the mass spectrometer was the same as that of the unesterified compound, esters (even when only those of palmitic acid were quantified) had, on average, half the concentration of PTX2sa in the digestive gland and were nearly absent from the remaining tissues (Figure 6).

The relationship between the pectenotoxins concentration and those of its derivatives was linear and statistically significant, both, in the digestive gland and in the remaining tissues (Supplementary Material). 

### 2.3. Anatomical Distribution of Toxins

**Figure 7:**
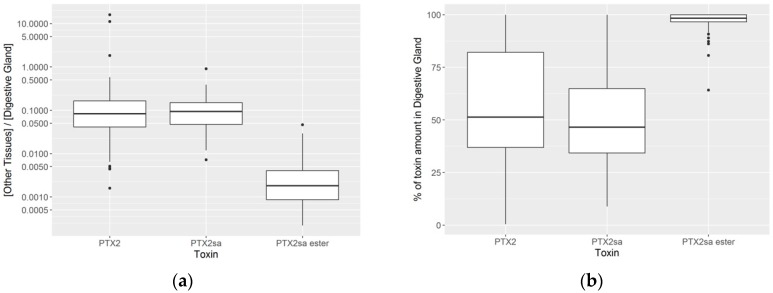
(**a**) Ratio between concentration of the toxins in digestive gland and other tissues (left panel) and (**b**) percentage of the total toxin burden in the digestive gland (right panel).

The concentrations of all toxins studied were much higher in the digestive gland than in the remaining tissues (Figure 7a). The concentrations of PTX2 and PTX2sa in the digestive gland were approximately 10-fold those in other tissues, but the difference was even more important for esters which were more than 300-fold more concentrated in the digestive gland (Figure 7a).

The amounts of PTX2 and PTX2sa were evenly distributed between the digestive gland and the remaining tissues (with slightly less PTX2sa in the remaining tissues) (Figure 7b), but nearly all esters were located in the digestive gland (with significant differences between esters and the other two toxins, but not between PTX2 and PTX2sa).

### 2.4. Dinophysis Abundance and Toxin Concentration

**Figure 8:**
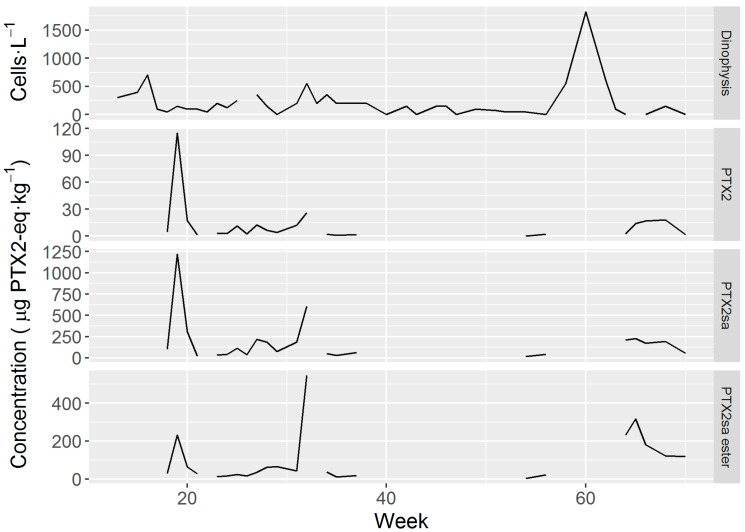
*Dinophysis acuminata* abundance and average weekly toxin concentrations in *M. donacium* in samples from Bahía Coquimbo. Periods not connected by lines correspond to weeks in which samples could not be obtained.

*Dinophysis* abundance was generally low, exceeding 500 cells L^−1^ on only a few occasions. The maximum weekly mean of cell concentration attained was 1825 cells L^−1^ (Figure 8). The time-course of toxin concentration of *M. donacium* in the digestive gland showed three main peaks, which took place at the same time for the PTX1, PTX2sa and PTX2sa esters. In general (when records of both, toxins and cells were available) the peaks of *D. acuminata* abundance and toxin concentration in surf clams did not coincide. 

### 2.5. Depuration Rates

**Figure 9:**
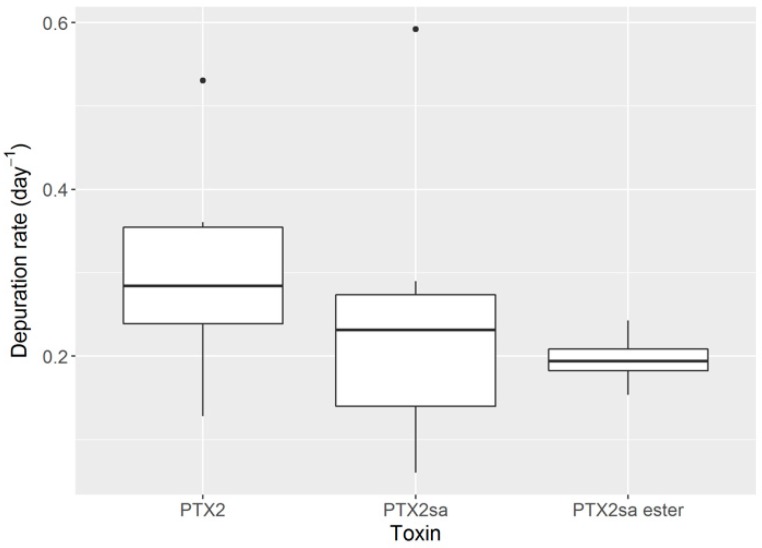
Estimated depuration rates for PTX2 and its derivatives.

The estimated depuration rates (Figure 9) were higher for PTX2 than for PTX2-sa and PTX2-sa esters. The average values were high at 0.3, 0.23 and 0.2 day^−1^, respectively.

## 3. Discussion

In Chile, the presence of *Dinophysis acuminata* has been described in several distinct geographical locations. In northern Chile this species has been described between 18 °S and 33 °S [18,19,20,21,22]. The taxonomic examination of specimens from phytoplankton net samples revealed that the main morphological features correspond to descriptions of this species given by Faust and Gulledge [23]. In relation to cell size, the length is consistent with measures given by Faust and Gulledge [23] (38–58 μm), Lebour [24] (38–51 μm), Dodge and Hart-Jones [25] (38–58 μm), Olenina et al. [26] (38–58 μm) and Reguera [27] (44–58 μm). However, our cells were larger than those reported by Sar et al. [28] (31.5–38 μm). In relation to cell width, our measures are consistent with the values reported by Olenina et al. [26] (30–38 μm), Faust and Gulledge [23] (30–40 μm) and Reguera [27] (24–43 μm).

*Dinophysis acuminata* was found to be persistent in the area, but without attaining high cell concentrations. This finding seems to be consistent with observations that the species is common in Northern Chile [18,19,20,21], but that it seldom results in market closures of fisheries or aquaculture products [18].

The toxin profiles observed in *M. donacium*, with a complete absence of toxins of the okadaic acid group, suggest that the lack of these toxins in *D. acuminata* from the area found in a bloom in 2005 by Blanco, Alvarez and Uribe [18], was not a special case but rather a general characteristic of this species in the area. The diversity of toxins and derivatives of the PTX group was very limited. Only PTX2 and some of its derivatives in the form of seco-acid and seco-acid esters were found, suggesting that the *Dinophysis* populations contain only PTX2, as PTX2-sa (as also seems to be the case on the Argentinian coast [29]) and its esters are formed by the action of the bivalve [16,17,30,31,32]. Apart from *Mytilus edulis* [14,17] and an “Australian clam” (cited by Doucet et al. [14] without specifying the species, but which was probably *Plebidonax deltoides* as high accumulations of PTX2sa had been found in this species in the area [7,33]), *Mesodesma donacium* is the first species in which esters of PTX2sa have been found, suggesting that this transformation could be general in molluscs. In other bivalve species, such as *Patinopecten yessoensis*, PTX2 undergoes an oxidation that yields PTX6 as the final product and PTX1 as intermediate one [34,35], but neither PTX6 nor PTX1 have been found in *M. donacium* which means that that oxidation route that generates these derivatives is not active in the species.

The fact that the observed peaks of cell abundance did not produce equivalent peaks of pectenotoxins in *M. donacium* suggests that there were substantial differences in toxin/cell among the different *D. acuminata* populations that developed throughout the sampled year. Other causes, such as differences in the availability of the toxic cells to the infaunal populations of the mollusks cannot be discarded. However, downwelling in the area (computed from the wind data of a meteorological station on-shore, data not shown)—the main process that could potentially regulate this availability— was not related to the toxin peaks in the clams.

The presence in *M. donacium* of PTX2, PTX2sa and PTX2sa esters suggests that PTX2 is transformed to PTX2sa and then to PTX2sa esters. The first step (PTX2 to PTX2sa) could take place in the gut, during the process of extracellular digestion of the ingested phytoplankton, as demonstrated by MacKenzie, Selwood and Marshall [16] for *Perna canaliculus* but it is possible that the transformation continues once PTX2 is inside the digestive cells as it has also been observed in vitro by treating PTX2 with homogenates [30,31,32] (or even in cells of other tissues). Esters of PTX2sa should be generated inside the cells as happens with other toxins, such as those of the OA group [13,36,37], brevetoxins [38], spirolides [39], gymnodimines [40], and other lipophilic compounds such as esteroids [41,42]. Very likely the mechanism is a transesterification similar to that found in OA [36,43]. It is clear that this process, in the case of *M. donacium*, only takes place in the digestive gland and not in other tissues, as their content in esters is marginal.

The fact that the apparent depuration rates from the digestive gland are lower as the compound required more transformation steps (PTX2 > PTX2-sa > PTX2-sa esters) can be explained in multiple ways. One possibility is that there were differences in the actual depuration rate of the compounds. A second possibility, which seems more likely, is that the biotransformations altered the estimated depuration rates because the losses by depuration of the transformed compound are increased by the amount of compound that is transformed, while those corresponding to the product compound are decreased by the same reason. A combination of the two processes could also take place. A more detailed study involving the analysis of these possibilities would be required to elucidate the precise cause of the observed differences.

The observed depuration rates, even though they are likely to be underestimates (because the cells rarely disappeared from the water), are relatively high in comparison with those of other lipophylic toxins as those in the OA group [44,45]. *Mesodesma donacium* seems to depurate PTXs much faster than Norwegian mussels and oysters, [46] with estimates of t_1/2_ (semidepuration time) of 6–13 days for PTX2 in mussels *Mytilus edulis* and oysters, while in this study they ranged from 2.3 to 3.1 days, for PTX2 and palmytoyl-PTX2sa, respectively. Notwithstanding this, in a previous study, the estimated depuration rates for PTX2 and PTX2sa from another mussel (*Mytilus galloprovincialis*), and the cockle *Cerastoderma edule*, were much higher, ranging from 0.6 to 1.1 and from 1 to 3 day^−1^, respectively (t_1/2_ 1.2–0.6 days and 0.7–0.2 days) [47]. Okadaic acid in the same studies was found to depurate from the bivalves at substantially lower rates [46,47].

These high depuration rates indicate that most of the accumulated toxins are likely to have been recently incorporated, and that the levels of these kinds of toxins in *M. donacium* are strongly dependent on the precise nature of the causative organisms.

## 4. Materials and Methods 

### 4.1. Area of Study, Phytoplankton Sampling, Quantification and Taxonomic Analyses

Phytoplankton samples were collected periodically (weekly when possible) in Bahía Coquimbo (29°51′ S, 71°16′ W) from 1 May 2009 to 28 April 2010, by means of vertical net hauls (20 μm mesh) and a 10 m hose, in order to obtain integrated samples of the entire water column. Bahía Coquimbo is a wide bay with a mean depth of 25 m, and is very dynamic, with typical surficial tidal currents of around 10 cm·s^−1^ and bottom currents between 4 and 13 cm·s^−1^ [48]. The bottom sediment is mostly sand and is sorted by depth, with the finest particles in the deepest locations [49], indicating a high dynamism in the shallow areas. A thermocline sometimes exists, at a depth of 10 m. The phytoplankton samples were obtained from the same location as those of *Mesodesma donacium*. Two aliquots were preserved—one with formaldehyde 4% (net hauls) and another with Lugol’s iodine (hose)—for taxonomic and quantitative analyses, respectively. Phytoplankton composition, including *D. acuminata* cells, were routinely identified using an Olympus IX71 epifluorescence inverted microscope and the method describe by Fritz and Triemer [50]. Phytoplankton and *D. acuminata* cells were quantified using the Utermöhl method, described by Hasle [51], using 10-mL sedimentation chambers with an Olympus IX71 inverted microscope.

### 4.2. Shellfish Sampling, Toxin Extraction and Hydrolysis

Shellfish samples were collected from 1 May 2009 to 28 April 2010 at the same station as the phytoplankton samples, from 10 to 15 m deep, by means of hookah diving. When possible, a weekly periodicity was maintained. Samples were homogenized and extracted with methanol 100% at a ratio of 1:4 (weight:volume). Extracts were clarified by centrifugation (10,000× *g*, 15 min) and then filtered through 0.20 μm Clarinert nylon syringe filters (13 mm diameter) (Agela technologies). 

In order to check the presence of derivatives of toxins of the okadaic acid group some extracts, selected because of their high PTX2 levels (which could be expected to be correlated with toxins of the OA group), were subjected to alkaline hydrolysis following the standard procedure of the EU Reference Laboratory for Marine Biotoxins [52].

### 4.3. Toxin Detection and Quantification

The toxins contained in the extracts were determined by HPLC-MS/MS, with a Thermo Accela chromatographic system (UHPLC) coupled to a Thermo TSQ Quantum Access Max by means of a HESI-II electrospray interface.

Basically, the chromatographic method by Regueiro et al. 2010 [53] was used, but modified in order to use a shorter column and to allow enough time for the elution, not only of the free toxins, but also of their acylated derivatives. Two chromatographic phases were used: A = 6.7 mM NH_4_OH in MilliQ water (Millipore); and B = 90% ACN with 6.7 mM NH_4_OH. First, the sample was injected into an online solid phase extraction (SPE) column (Phenomenex Security Guard 4 × 2 mm with phase Gemini-NX C18 (AJO-8367) in an isocratic flow of 90% A and 10% B, while the chromatographic column was kept at 80% A. After 1.5 min the system flow was switched (with a Rheodyne 2-position 6-way valve) and the content of the SPE column started to elute to the chromatographic column (Phenomenex Gemini-NX C18 50 × 2 mm 3 µm). The phase B percentage was raised in a linear manner until reaching 90% at min 3.85 and maintained at that concentration until min 8.25 when the initial conditions were put in place again and maintained until min 10.5. At min 7.5 the Rheodyne valve was switched again in order to equilibrate the SPE column for the next injection. For detailed analysis of PTX2sa acyl-derivatives the chromatographic gradient was modified by extending it for 3 additional minutes.

The mass spectrometer was operated in positive and negative ionization mode using the following settings: Spray Voltage Positive 3500 V, Negative 3000 V, Sheath Gas Pressure 50, Aux Gas Flow: 5; Vaporizer Temperature: 110; Capillary Temperature 360; Collision Gas Pressure (mTorr): 1.5. For identification and quantification, the transitions given in Table 1 were used.

The toxin concentration in the extracts was quantified by comparing the area or the peaks obtained in the chromatograms with those of certified reference materials obtained from Laboratorio CIFGA, Spain and the NCR, Canada. When those materials were not available—as was the case for PTX2 seco-acid and esters of PTX2 seco-acid—a relative quantification was carried out using the signal of PTX2 as reference.

### 4.4. Estimation of Depuration Rates

Rough estimates of depuration rates were obtained by using concentration values in two consecutive weeks based on the following selection criteria: (a) that the first observation had a high concentration value; (b) that the *D. acuminata* abundance in the following week was low; and (c) there was no substantial increase of toxin concentration in the third week. This approach would yield underestimated values of the depuration rate as some toxin uptake had taken place during the period for which depuration was estimated. It was assumed that depuration followed an exponential decrease, and the rate for each period was computed as Ln[Tox]_week0_ − [Tox]_week1_)/7 days and was expressed as day^−1^.

### 4.5. Statistical Analysis

Regression, correlation and ANOVA analyses were carried out with *R* [54]. Descriptive statistical plots were built with the ggplot2 package [55]. 

## Figures and Tables

**Table 1:** Transitions used to identify and quantify the compounds studied (CE = Collision Energy (V)).

Reference	Parent	Product	CE
***PTXs method***			
OA_DTX-2	803.5	255.2	48
OA_DTX-2	803.5	563.4	43
DTX-1	817.5	255.2	48
DTX-1	817.5	563.5	43
PTX-2	876.5	805.5	23
PTX-2	876.5	823.5	21
PTX1	892.5	839.5	23
PTX6	906.5	853.5	23
PTX12	874.5	821.5	23
PTX2sa	894.5	823.5	21
PTX2sa	894.5	805.5	21
PTX11sa	910.5	179.2	50
PTX11sa	910.5	137.2	50
C16-PTX2sa C_33,37_	1132.6	823.5	23
C16-PTX2sa C_11_	1132.6	1061.5	23
***Acyl derivatives method***			
C14:0-PTX2sa	1104.6	823.5	23
C15:0-PTX2sa	1118.6	823.5	23
C16:1-PTX2sa	1130.6	823.5	23
C16:0-PTX2sa	1132.6	823.5	23
C17:1-PTX2sa	1144.6	823.5	23
C17:0-PTX2sa	1146.6	823.5	23
C18:5-PTX2sa	1150.6	823.5	23
C18:4-PTX2sa	1152.6	823.5	23
C18:3-PTX2sa	1154.6	823.5	23
C18:2-PTX2sa	1156.6	823.5	23
C18:1-PTX2sa	1158.6	823.5	23
C18:0-PTX2sa	1160.6	823.5	23
C20:5-PTX2sa	1178.6	823.5	23
C20:4-PTX2sa	1180.6	823.5	23
C20:2-PTX2sa	1184.6	823.5	23
C20:1-PTX2sa	1186.6	823.5	23
C20:0-PTX2sa	1188.6	823.5	23
C22:6-PTX2sa	1204.6	823.5	23

## Supplementary Materials

The following are available online at http://www.mdpi.com/2072-6651/10/8/314/s1, Raw data of toxin concentration in the extract. Raw data of *Dinophysis acuminata* cell counts. Figure S1: Relationships between the PTX2 derivatives.
