# Supplementary figures and images for: Accumulation and Biotransformation of Dinophysis Toxins by the Surf Clam Mesodesma donacium
Source: Toxins (Basel). 2018 Aug 4;10(8):314. doi: 10.3390/toxins10080314 (PMC6115731; doi:10.3390/toxins10080314)

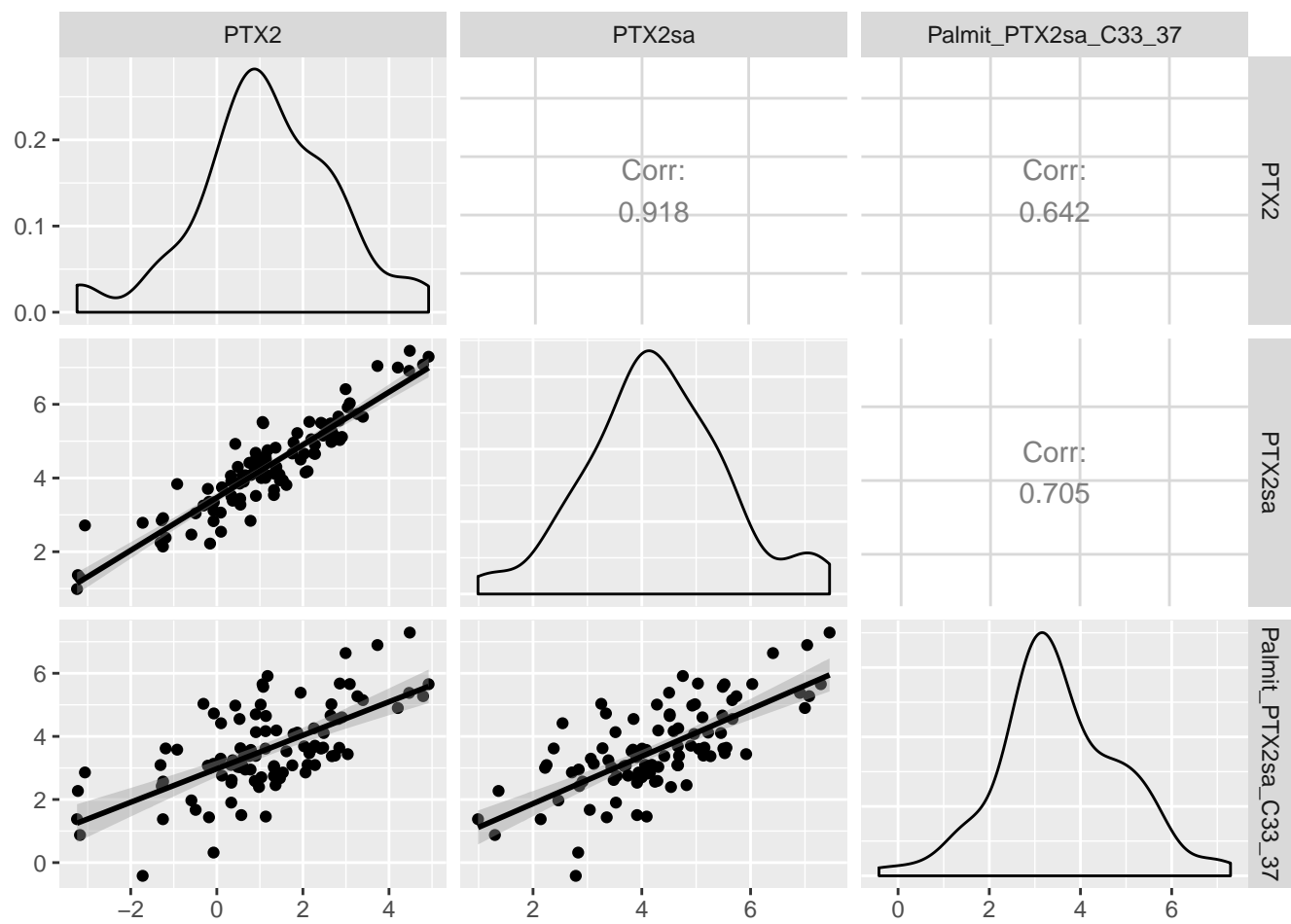

Supplement: Supplementary file 1 [file toxins-10-00314-s001.zip › GRregresGeneral.pdf]
